# Pregnancy outcomes in patients with acute kidney injury during pregnancy: a systematic review and meta-analysis

**DOI:** 10.1186/s12884-017-1402-9

**Published:** 2017-07-18

**Authors:** Youxia Liu, Xinxin Ma, Jie Zheng, Xiangchun Liu, Tiekun Yan

**Affiliations:** 1 Department of Nephrology, General Hospital of Tianjin Medical University, NO. 154, Anshan road, Heping District, Tianjin, China; 2 Division of Nephrology, Department of Medicine, The Third Affiliated Hospital of Sun Yat-Sen University, Guangzhou, China; 3 Radiology Department, General Hospital of Tianjin Medical University, Tianjin, China; 4 Department of Nephrology, The Second Hospital of Shandong University, Shandong University, Jinan, China

**Keywords:** Acute kidney injury, Pregnancy outcomes, Kidney outcome, Meta-analysis

## Abstract

**Background:**

Presently, the matter of pregnancy outcomes of patients with pregnancy related AKI (PR-AKI) were disputed. Thus, we conducted a meta-analysis to evaluate the impact of PR-AKI on pregnancy outcomes.

**Method:**

We systematically searched MEDLINE, Embase, VIP, CNKI and Wanfang Databases for cohort or case-control studies in women with PR-AKI and those without AKI as a control group to assess the influence of PR-AKI on pregnancy outcomes and kidney outcome. Reduction of odd ratio (OR) was calculated by a random-effects model.

**Results:**

One thousand one hundred fifty two articles were systematically reviewed, of those 11 studies were included, providing data of 845 pregnancies in 834 women with PR-AKI and 5387 pregnancies in 5334 women without AKI. In terms of maternal outcomes, women with PR-AKI had a greater likelihood of cesarean delivery (OR, 1.49; 95% confidence interval [CI], 1.37 to 1.61), hemorrhage (1.26; 1.02 to 1.56), HELLP syndrome (1.86; 1.41 to 2.46), placental abruption (3.13; 1.96 to 5.02), DIC (3.41; 2.00 to 5.84), maternal death (4.50; 2.73 to 7.43), but had a lower risk of eclampsia (0.53; 0.34 to 0.83). Women with PR-AKI also had a longer stay in ICU (weighted mean difference, 2.13 day [95% CI 1.43 to 2.83 day]) compared with those without PR-AKI. As for fetal outcomes, higher incidence of stillbirth/perinatal death (3.39, 2.76 to 4.18), lower mean gestational age at delivery (−0.70 week [95% CI -1.21 to −0.19 week]) and lower birth weight (−740 g [95% CI -1180 to 310 g]) were observed in women with PR-AKI. The occurrence of kidney outcome, defined as ESRD requiring dialysis, in women with PR-AKI was 2.4% (95% CI 1.3% to 4.2%).

**Conclusions:**

PR-AKI remains a grave complication and has been associated with increased maternal and fetal mortality.

**Electronic supplementary material:**

The online version of this article (doi:10.1186/s12884-017-1402-9) contains supplementary material, which is available to authorized users.

## Background

The incidence of pregnancy related acute kidney injury (PR-AKI) has decreased markedly worldwide during the past 50 years, probably due to improvement of obstetric and prenatal care as well as decline in rate of illegal abortion [1, 2]. However in recent years, the rate of PR-AKI appears to be on the rise even in some developed countries [3, 4]. Even though the incidence of PR-AKI has been declining, it remains a serious problem due to its association with significant adverse maternal and fetal outcomes [5–8]. According to some studies, the rates of maternal mortality and fetal loss in patients with PR-AKI have risen to 30 and 60% [8].

In the past, AKI was considered to be a completely reversible syndrome [9], however, in recent years, several studies have indicated that AKI may increase the risk of developing chronic kidney disease (CKD), resulting in permanent kidney damage [10–12]. Although some amount of research effort gone into PR-AKI, it is often difficult to accurately evaluate the risk of pregnancy outcomes and kidney outcomes in PR-AKI women due to small sample sizes.

Thus, we undertook a systematic review and meta-analysis to evaluate the risk of adverse pregnancy outcomes and end-stage renal disease (ESRD) in women with PR-AKI versus those without AKI.

## Methods

### Search strategy and study selection

We performed a systematic review of the literature based on the approach recommended by the preferred reporting items for systematic reviews and meta-analysis (PRISMA) statement for reporting meta-analysis [13]. Referenced electronic literature from 1986 to April 2016 was available in MEDLINE, Embase, three Chinese databases (Chongqing VIP Chinese Science and Technology Periodical Database (VIP), China National Knowledge Infrastructure (CNKI) and Wanfang database). The search strategy used the Medical Subject Heading and text key words:“pregnancy” and “pregnant” combined with all spellings of “acute renal insufficiency”,“acute kidney injury”, “acute renal failure”. Cohort or case-control studies that reported pregnancy outcomes and kidney outcomes in women with PR-AKI and those without AKI as a control group to assess the influence of PR-AKI on pregnancy outcomes and kidney outcome, without language restrictions were included (Additional file 1: Text S1). Women with pre-existing CKD were excluded from the study. As only previously published studies were used no ethical review was required.

### Data extraction and quality assessment

The following information was retrieved from the studies included: definition of AKI, country in which the study was performed, number of patients, number of pregnancies, patient age, median/mean serum creatine (Scr). Pregnancy outcomes, including maternal outcomes and fetal outcomes, were also extracted. Maternal outcomes cover cesarean rate, eclampsia, hemorrhage during and after delivery, HELLP syndrome (hemolysis, elevated liver enzymes, and low platelet), placental abruption, disseminated intravascular coagulation (DIC), maternal death and length of ICU stay. On the other side, fetal outcomes were defined in terms of gestational age (i.e., how many weeks before 40 weeks’ gestation), birth weight and stillbirth/perinatal death. Data on kidney outcome was also obtained, defined as ESRD requiring dialysis. The literature search, data extraction and quality assessment (Grading of Recommendations Assessment, Development and Evaluation system) [14] were performed independently by two investigators (YXL and XXM) using a standardized approach. Any discrepancy between these two investigators was adjudicated by a third reviewer (JZ).

### Statistical analysis

Continuous variables such as length of ICU/hospitalization stay and birth weight were assessed by weighted mean difference between groups. The odds ratio (OR) and 95% confidence interval (CI) for each outcome was calculated before pooling by the random-effects model. Heterogeneity across the included studies was analyzed using the I^2^ to describe the percentage of variability (greater than 50% as evidence of a significant level) [15]. Subgroup analysis was conducted to assess the effect of adjustment for the key covariates. Potential publication bias was assessed with the Begg’s test and represented graphically with Begg funnel plots of the natural log of the OR versus its standard error (SE). A two-tailed *P* value less than 0.05 was considered statistically significant. All statistical analyses were performed using Stata 12.0, Review Manager 5.1 or Meta Analyst Beta 3.13 software.

## Results

**Fig. 1:**
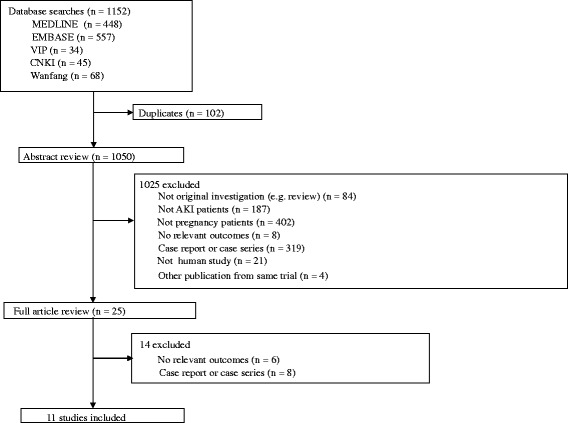
Process for identifying studies eligible for the meta-analysis

The literature search yielded 1152 articles, with 11 studies identified according to the inclusion criteria (Fig. 1) [8, 16–25]. Table 1 summarized the characteristics of these included studies. These studies were performed between 2004 to 2015 with sample sizes ranging from 38 to 3553, and numbers of pregnancies ranged from 41 to 3555. Most of the studies obtained were retrospective study or single-center experience. Four of the studies included were conducted in the ICU, however the other seven were conducted in the Obstetrics or Nephrology department. Two studies were conducted in Morocco, one from Tunisia, one from French, one from Turkey and the other six from China (Table 1). The definition of PR-AKI across these studies varied considerably, one diagnosed according to the RIFLE classification; four according to the Acute Kidney Injury Network (AKIN) classification; Five studies defined PR-AKI by the level of Scr, of which two enrolled pregnant women with Scr > 0.8 mg/dL, one with Scr > 1.0 mg/dL, one with Scr > 1.6 mg/dL and one with Scr > 1.2 mg/dL and/or oliguria <400 mL/24 h; One study used two criteria to enroll pregnant women with Scr > 0.8 mg/dL or meeting AKIN classification. All of these studies reported pregnancy outcomes in women with PR-AKI and without AKI. Only one study described the kidney outcome in both pregnant women with PR-AKI and those without AKI. Seven studies reported only kidney outcome in women with PR-AKI.

**Table 1:** Characteristics of included studies

Study	Country	Sample size of patients^a^	Sample size of pregnancies^a^	Definition of AKI	Mean Scr^b^ (umol/L)	Follow-up (y)	Mean Maternal Age (y)	Primiparous (%)	No.of ESRD (%)	No.of Maternal Mortality Events (%)	No.of Fetal Mortality Events (%)
Bentata 2012 [8]	Morocco	137 (46/91)	137 (46/91)	RIFLE	176/70^c^	3	31.2	69 (50)	2 (1.5)	17 (12.4)	48 (35.0)
Bouaziz 2013 [16]	Tunisia	550 (313/237)	550 (313/237)	Scr > 0.8 mg/dL	150/53	17	33.8	264 (48)	2 (0.4)	33 (6.0)	
Liu 2015 [20]	China	38 (22/16)	41 (24/17)	1.AKIN; 2. Scr > 0.8 mg/dL	165/72^c^	9	34.3	23 (61)	1 (2.6)	0 (0)	8 (19.5)
Zhu 2011 [19]	China	494 (40/454)	533 (40/493)	AKIN	216/68	1	33.9	222 (45)			80 (15.0)
Mei 2012 [24]	China	600 (90/510)	606 (90/516)	AKIN		4	33.4	440 (73)	1 (0.2)		51 (8.4)
Zeng 2011 [22]	China	174 (54/120)	174 (54/120)	AKIN		3	34	125 (72)	1 (0.6)		28 (16.1)
Chen 2011 [23]	China	3553 (85/3468)	3555 (85/3470)	Scr > 0.8 mg/dL		5	38.1				164 (4.6)
Jonard 2014 [18]	France	182 (68/114)	193 (74/119)	Scr > 1.0 mg/dL		5	36 ^c^		3 (1.6)		
Zhang 2011 [25]	China	130 (50/80)	130 (50/80)	AKIN		1	38.2				19 (14.6)
Mjahed 2004 [21]	Morocco	178 (46/132)	178 (46/132)	Scr > 1.5 mg/dL	343/<140	7	35.1	112 (63)	1 (0.6)	27 (15.2)	
Gul 2004 [17]	Turkey	132 (20/112)	135 (23/112)	Scr > 1.2 mg/dL and/or oliguria <400 mL/24 h	274/70	1	32.6		0 (0)		20 (14.8)

*Abbreviations*: *AKI* Acute kidney injury, *Scr* serum creatinine, *ESRD* End stage renal disease

^a^Expressed as total number of patients (number in PR-AKI group/number in control group)

^b^Expressed as the mean of Scr in the PR-AKI group / the mean in the control group

^c^Median

### Pregnancy outcomes

**Fig. 2:**
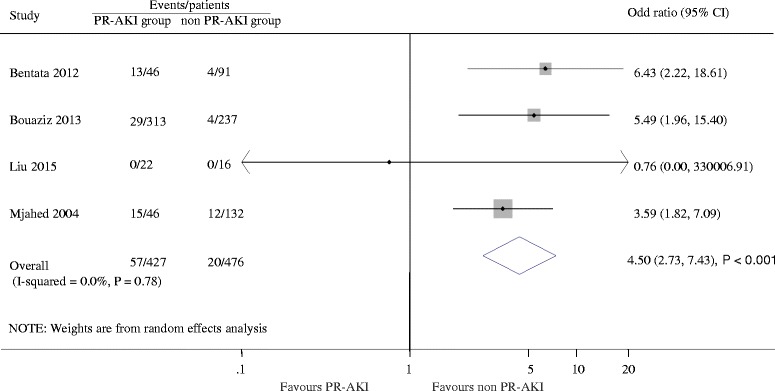
Hazard ratios of maternal death for pregnant women with versus without acute kidney injury

Pregnancy outcomes included maternal and fetal outcomes. Eight studies reported maternal outcomes. The maternal death and length of ICU stay were reported in four and five studies, respectively. Of the 427 women with PR-AKI there were 57 deaths (13.3%) and 20 deaths occurred in 476 women without AKI (4.2%). AKI during pregnancy was associated with a higher risk of maternal death event (OR 4.50; 95% CI 2.73 to 7.43, *P* < 0.001), with no evidence of heterogeneity (I^2^ = 0.0%, *P* = 0.78, Fig. 2). In addition, patients with PR-AKI had a longer stay in ICU (weighted mean difference, 2.13 day [95% CI 1.43 to 2.83 day], *P* < 0.001; I^2^ = 68%, *P* = 0.01, Additional file 2: Figure S1) than patients without PR-AKI. Seven studies reported 247 cesarean deliveries in 337 women with PR-AKI and 1574 cesarean deliveries of the 4013 women without AKI, producing a 1.49 fold (95% CI 1.37 to 1.61, *P* < 0.001) higher likelihood in women with PR-AKI compared with those without, with evidence of extensive heterogeneity (I^2^ = 95.0%, *P* < 0.001; Additional file 3: Figure S2). In order to diminish the heterogeneity, a subgroup analysis was performed based on country for comparison which led to a nearly 20.0% decrease of I^2^ (Additional file 4: Figure S3). Compared with the controls, women with PR-AKI had a higher incidence of hemorrhage (1.26; 1.02 to 1.56, *P* = 0.03; I^2^ = 63.1%, *P* = 0.04), HELLP syndrome (1.86; 1.41 to 2.46, *P* < 0.001; I^2^ = 70.3%, *P* = 0.04), placental abruption (3.13; 1.96 to 5.02, *P* < 0.001; I^2^ = 0.0%, *P* = 0.92), DIC (3.41; 2.00 to 5.84; *P* < 0.001; I^2^ = 0.0%, *P* = 0.85), but had a lower risk of eclampsia (0.53; 0.34 to 0.83, *P* = 0.006; I^2^ = 0.0%, *P* = 0.83). The details are shown in Additional file 5: Figure S4. Since only few studies mentioned maternal outcomes, there was insufficient data to allow analysis of heterogeneity of these outcomes.

**Fig. 3:**
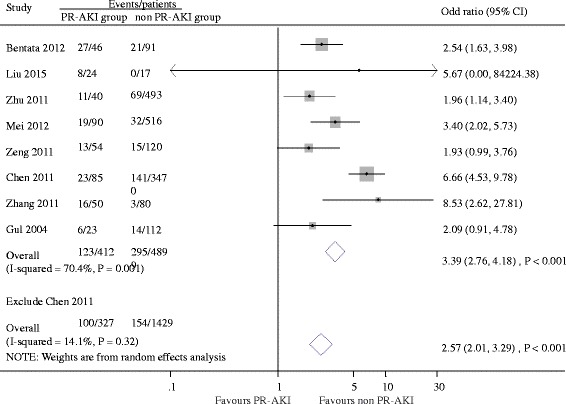
Hazard ratios of fetal death for pregnant women with versus without acute kidney injury

**Fig. 4:**
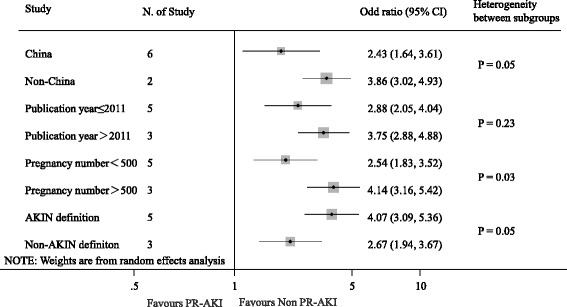
Subgroup analysis of the hazard ratios of fetal death for pregnant women with versus without acute kidney injury

Ten studies reported fetal outcomes. Gestational age and birth weight were evaluated in ten and five studies respectively. The results showed that PR-AKI was associated with lower mean gestational age at delivery (weighted mean difference, −0.70 week [95% CI -1.21 to −0.19 week], *P* = 0.007; I^2^ = 97.0%, *P* < 0.001, Additional file 6: Figure S5) and lower baby birth weight (weighted mean difference, −740 g [95%CI −1180 to −310 g], *P* < 0.001; I^2^ = 95.0%, *P* < 0.001, Additional file 7: Figure S6). Overall, there were 123 stillbirth/perinatal deaths of the 412 pregnancies with PR-AKI (29.8%) and 295 deaths among 4899 pregnancies without AKI (6.0%). PR-AKI increased the risk of stillbirth/perinatal death in the pooled analysis (OR 3.39; 95% CI 2.76 to 4.18, *P* < 0.001), with evidence of extensive heterogeneity (I^2^ = 70.4%, *P* = 0.001, Fig. 3). We further explored the reasons for heterogeneity in stillbirth/perinatal death. The pregnancy number was partly accounted for the heterogeneity (Fig. 4). This analysis was dominated by a large Chinese study (3555 pregnancies; accounting for 29.2% of the weight). Exclusion of this study did not eliminate the higher risk of stillbirth/perinatal death (OR, 2.57; 95% CI 2.01 to 3.29; *P* < 0.001) associated with PR-AKI but dropped heterogeneity to 14.1% (I^2^ = 14.1%, *P* = 0.32) (Fig. 3).

### Kidney outcome

Only one study provided kidney outcome in women with PR-AKI and those without AKI, with no significant difference between these two groups (0% vs. 0%). Data regarding the effects of pregnancy on kidney outcome in women of PR-AKI were available from eight studies with 659 women, among which 11 events were observed. The pooled results, based on a random effects model, showed 2.4% (95% CI 1.3% to 4.2%) women with AKI during pregnancy progressed to ESRD and needed long-term dialysis.

### Risk of bias within studies

The GRADE evaluation indicated that maternal and fetal mortality had low-quality evidence (Additional file 8: Table S1). Funnel plot of Begg’s test was used to show evidence of the publication bias, and found there were no bias for maternal and fetal mortality among studies (Begg’s test, *P* = 0.73 and *P* = 0.90, respectively) (Additional file 9: Figure S7 and Additional file 10: Figure S8).

## Discussion

Pregnancy related acute kidney injury has a significant impact on both maternal and fetal morbidity and mortality. Unfortunately, the exact definition of pregnancy related acute kidney injury employed in different studies didn’t reach a consensus and lacked sensitivity. Consequently, it’s difficult to accurately estimate the incidence of pregnancy related acute kidney injury and quantify its influence on mortality. Due to the physiological changes and increase in glomerular filtration rate, a reduction of the serum creatinine during pregnancy makes the early and accurate diagnosis of pregnancy related acute kidney injury more difficult [2]. The small sample sizes and limited clinical data available in published reports precluded pooled estimates of the effects of pregnancy related acute kidney injury on pregnancy outcomes and kidney outcomes. However, the widely use of AKIN and RIFLE classification and staging systems in recent years gives an opportunity to generate more valid and generalizable pooled estimates.

In current meta-analysis, 11 included studies of nearly 6000 pregnancies analyzed the relationship between pregnancy related acute kidney injury and pregnancy outcomes. It was confirmed that pregnancy related acute kidney injury was associated with higher risk of maternal death (4.5 fold), cesarean delivery (1.49 fold), and stillbirth/perinatal death (3.39 fold). Overall, women with pregnancy related acute kidney injury stayed longer time in ICU and delivered lower birth weight babies than those without acute kidney injury. The maternal mortality was higher than that in normal gravida without acute kidney injury, so well as the rate of fetal loss. The maternal mortality of 13.3% in women with pregnancy related acute kidney injury in our study was similar to some of the studies reported by Kabbali et al. (11.4%) [26], Godara et al. (15.7%) [5], Hassan et al. (16.2%) [27] from developing countries. The fetal mortality was observed in 29.8% of the pregnancies, which was higher than controls (6.0%). Multivariate Logistic regression analysis showed that acute kidney injury was the main independent factor associated with maternal mortality in Bentata’s study [8]. Other researchers found the advanced stages of acute kidney injury are associated with high maternal mortality in patients with pregnancy related acute kidney injury [16, 28, 29]. The rate of maternal mortality was significantly high, even in patients without acute kidney injury it was recorded to be as high as 4.2%. In total, three of the four studies which provided data of maternal death conducted in ICU in developing countries, patients including those without acute kidney injury admitted to the ICU for serious complications of pregnancy such as preeclampsia, eclampsia, hemorrhagic shock, hemolysis, elevated liver enzymes, and low platelet (HELLP syndrome), severe sepsis and acute pregnancy fatty liver. The severity of the profile is related to poor prenatal care, inadequate healthcare structures, and to the absence of appropriate management of serious intrapartum complications. The high rate of fetal mortality may also be due to underlying comorbidities as well as iatrogenic early termination of pregnancy. Further subgroup analysis did not show a significant modifying effect of fetal mortality (ure 4). The high mortality in patients with pregnancy related acute kidney injury indicates that there is substantial scope for improvement in prenatal care and obstetrical services to prevent such serious complications.

Several pregnancy-related disorders such as hemorrhage, eclampsia, hemolysis, elevated liver enzymes, and low platelet (HELLP syndrome), abruption placenta and disseminated intravascular coagulation (DIC) were more commonly seen in pregnant women with acute kidney injury. Acute kidney injury is a rare complication of pregnancy and typically occurs in healthy women who acquire a major pregnancy-related medical condition such as hemorrhage, eclampsia, hemolysis, elevated liver enzymes, and low platelet (HELLP syndrome), which are the leading causes of acute kidney injury during pregnancy. Paradoxically, in our study, the rate of eclampsia was found to be 24.6% in women without pregnancy related acute kidney injury, which was higher than those with pregnancy related acute kidney injury (13.4%). This analysis was dominated by the study of Bentata et al., which noted that hypertensive disorders were the main contributing etiology of obstetric acute kidney injury and the incidence for preeclampsia and eclampsia was up to 60.9% in Morocco. However, owing to lack of sufficient baseline data in our study, further analysis of these disorders was impossible. Delays in diagnosis and inappropriate management of these pregnancy disorders may result in irreversible renal damage. Careful patient counseling and close follow-up in a tertiary care center, where cooperation between nephrologists and obstetricians is available, is highly recommended.

Whether acute kidney injury during pregnancy would increase the potential risk of chronic kidney disease remained inconclusive. A case-control study from Gul et al. showed no significant difference in kidney outcome between pregnant women with and without acute kidney injury [17]. In a retrospective study conducted in women with acute kidney injury treated with dialysis during pregnancy over a 15-year period in Canada, Hildebrand and colleagues observed 3.9% of patients remained dialysis dependent 4 months after delivery [6]. One study showed that acute kidney injury was an independent risk factor for end-stage renal disease, which suggested that episodes of acute kidney injury may cause chronic renal inflammation and subsequent fibrosis which resulted in long-term renal dysfunction [30]. Using US Renal Data System, Ishani and colleagues observed the incidence for end-stage renal disease following acute kidney injury among elderly patients was 4.08% per year during 2 years of follow-up [31]. A systematic overview published in 2012 that included 13 cohort studies and more than 1400,000 individuals performed by Coca et al., found the pooled incidence of end-stage renal disease was up to 8.6 per 100 person-years and patients with acute kidney injury had a higher risk of developing end-stage renal disease than patients without acute kidney injury [11]. In a subsequent study, Wald and colleagues reported the incidence rate for end-stage renal disease was only 1.78% over 10 years of follow-up in patients who had non-dialysis-requiring acute kidney injury by using provincial health registry data from Ontario, Canada [32]. In our study, 2.4% of women with acute kidney injury during pregnancy progressed to end-stage renal disease and had to receive long-term dialysis. Due to most studies lacking the presence of a control group, small sample sizes and limited follow-up time, these precluded us from accurately evaluating the risk of acute kidney injury on long-term renal function in pregnant women. More cohort studies and case-control studies are required to evaluate whether acute kidney injury causes permanent damage to long-term kidney function in pregnant women.

Strength of this systematic review and meta-analysis lied in the instruction significance for clinical question, large volume of data included and rigorous methodology used. However, our study had several limitations. First is the limitation of using a variety of diagnostic criteria for acute kidney injury and the lack of recognition of acute kidney injury in pregnancy, which would affect the severity of disease and complications each study subsequently reported. Second, biases towards the reported rate of death may have ensued, as data which yielded the most important results came from patients in ICU who developed serious complications during pregnancy. And it is highly possible that we may miss some mild patients who developing an acute and rapidly resolving mild renal failure in the postpartum period. Finally, the limitations of small sample sizes stemming from the exclusion of many studies discussing kidney outcomes due to them are lacking a control group of pregnant women without acute kidney injury. As a consequence, it was difficult to obtain a definitive conclusion about the effect of pregnancy related acute kidney injury on kidney disease progression in pregnant women.

## Conclusion

Pregnancy related acute kidney injury remains a grave complication that has been associated with relatively higher risk of maternal and fetal mortality. Further studies are greatly required in order to develop a more sensitive diagnosis standard for pregnancy related acute kidney injury and provide definitive evidence regarding the impact of PR-AKI on long-term kidney function.

## Additional files

Additional file 1: Text S1 Search strategy. (DOCX 14 kb)

Additional file 2: Figure S1. Length of ICU/hospitalization stay (day) in pregnant women with versus without acute kidney injury. (PPTX 57 kb)

Additional file 3: Figure S2. Hazard ratios of cesarean delivery for pregnant women with versus without acute kidney injury. (PPTX 68 kb)

Additional file 4: Figure S3. Hazard ratios of cesarean delivery for pregnant women with versus without acute kidney injury by country. (PPTX 73 kb)

Additional file 5: Figure S4. Hazard ratios of eclampsia, HELLP syndrome, placental abruption, and DIC for pregnant women with versus without acute kidney injury. (PPTX 92 kb)

Additional file 6: Figure S5. Gestational age at delivery in pregnant women with versus without acute kidney injury. (PPTX 84 kb)

Additional file 7: Figure S6. Birth weight in pregnant women with versus without acute kidney injury. (PPTX 64 kb)

Additional file 8: Table S1. Grade quality of evidence (DOCX 90 kb)

Additional file 9: Figure S7. Funnel plot with pseudo 95% confidence limits of maternal mortality among included studies. (PPTX 42 kb)

Additional file 10: Figure S8. Funnel plot with pseudo 95% confidence limits of fetal mortality among included studies. (PPTX 42 kb)

## Abbreviations

AKIN: Acute Kidney Injury Network
CI: confidence interval
CKD: chronic kidney disease
DIC: disseminated intravascular coagulation
ESRD: end-stage renal disease
HELLP: hemolysis, elevated liver enzymes, and low platelet
OR: odd ratio
PR-AKI: pregnancy related acute kidney injury
PRISMA: preferred reporting items for systematic reviews and meta-analysis
Scr: serum creatine
