# Supplementary material for: Pregnancy outcomes in patients with acute kidney injury during pregnancy: a systematic review and meta-analysis
Source: BMC Pregnancy Childbirth. 2017 Jul 18;17:235. doi: 10.1186/s12884-017-1402-9 (PMC5516395; doi:10.1186/s12884-017-1402-9)
Supplement: Supplementary file 1 — Text S1 Search strategy. (DOCX 14 kb) [file 12884_2017_1402_MOESM1_ESM.docx]

| **Additional file 1**  **Search strategy**  **Medline (ovid)**   1. exp pregnancy/ 2. exp conception/ 3. exp gestation/ 4. exp childbirth/ 5. exp foetus/ 6. exp fetus/ 7. exp Newborn/ 8. pregnan$.tw. 9. gestation$.tw. 10. (new adj5 born).tw. 11. labo$.tw. 12. fetal$.tw. 13. 1 or 2 or 3 or 4 or 5 or 6 or 7 or 8 or 9 or 10 or 11 or 12 14. exp Clinical Trial/ 15. exp Cohort Studies/ 16. exp Case-control Studies/ 17. Prospective Studies/ 18. Retrospective Studies/ 19. Longitudinal Studies/ 20. Follow-up Studies/ 21. cohort$.tw 22. (case$ adj5 control$).tw. 23. (longitudinal$ adj5 stud$).tw. 24. (follow$ adj5 stud$).tw. 25. (retrospective$ adj5 stud$).tw. 26. (prospective$ adj5 stud$).tw. 27. 14 or 15 or 16 or 17 or 18 or 19 or 20 or 21 or 22 or 23 or 24 or 25 or 26 28. exp acute renal failure/ 29. exp acute kidney disease/ 30. exp acute kidney injury / 31. exp acute renal insufficiency / 32. acute renal$.tw. 33. acute kidney$.tw. 34. 28 or 29 or 30 or 31 or 32 or 33 or 34 or 35 35. 13 and 27 and 34   EMBASE   1. pregnancy 2. conception 3. gestation 4. fetus 5. foetus 6. labor 7. newborn 8. new NEAR/5 born 9. childbirth 10. labour* 11. pregnan* 12. 1 or 2 or 3 or 4 or 5 or 6 or 7 or 8 or 9 or 10 or 11 13. clinical trial 14. cohort study 15. case control study 16. prospective study 17. retrospective study 18. longitudinal study 19. follow up study 20. prospective* NEAR/5 stud* 21. retrospective* NEAR/5 stud* 22. follow* NEAR/5 stud* 23. longitudinal* NEAR/5 stud* 24. case* NEAR/5 control* 25. 13 or 14 or 15 or 16 or 17 or 18 or 19 or 20 or 21 or 22 or 23 or 24 26. acute renal failure 27. acute kidney disease 28. acute kidney injury 29. acute renal insufficiency 30. acute renal* 31. acute kidney* 32. 26 or 27 or 28 or 29 or 30 or 31 33. 12 and 25 and 32 |  |  |
| --- | --- | --- |
|  |  |  |
