# Supplementary material for: Pregnancy outcomes in patients with acute kidney injury during pregnancy: a systematic review and meta-analysis
Source: BMC Pregnancy Childbirth. 2017 Jul 18;17:235. doi: 10.1186/s12884-017-1402-9 (PMC5516395; doi:10.1186/s12884-017-1402-9)
Supplement: Supplementary file 2 — Length of ICU/hospitalization stay (day) in pregnant women with versus without acute kidney injury. (PPTX 57 kb) [file 12884_2017_1402_MOESM2_ESM.pptx]

## Slide 1
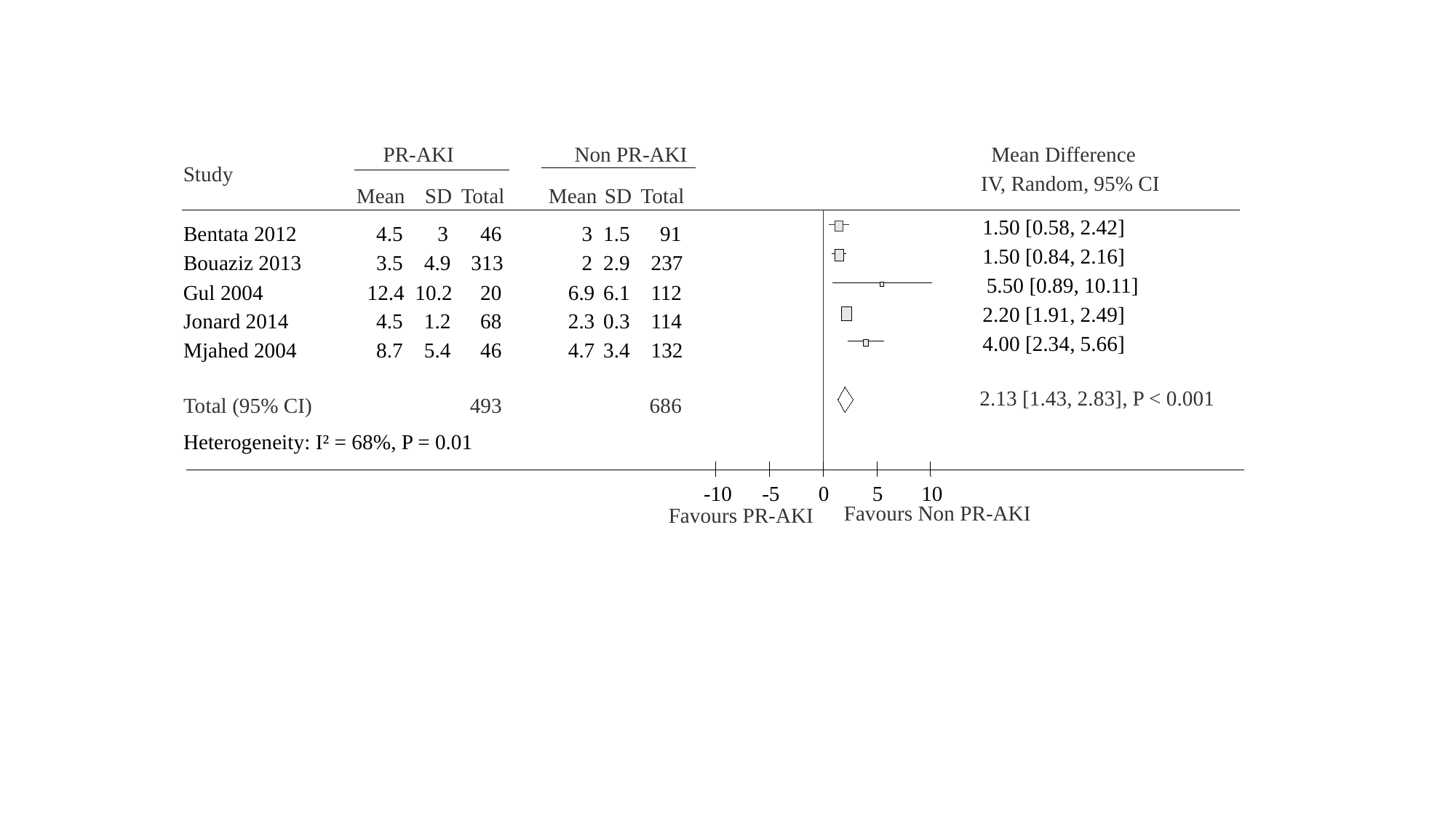

PR-AKI
Non PR-AKI
Mean Difference
Study
IV, Random, 95% CI
Mean
SD
Total
Mean
SD
Total
1.50 [0.58, 2.42]
Bentata 2012
4.5
3
46
3
1.5
91
1.50 [0.84, 2.16]
Bouaziz 2013
3.5
4.9
313
2
2.9
237
5.50 [0.89, 10.11]
Gul 2004
12.4
10.2
20
6.9
6.1
112
2.20 [1.91, 2.49]
Jonard 2014
4.5
1.2
68
2.3
0.3
114
4.00 [2.34, 5.66]
Mjahed 2004
8.7
5.4
46
4.7
3.4
132
2.13 [1.43, 2.83], P < 0.001
Total (95% CI)
493
686
Heterogeneity: I² = 68%, P = 0.01
-10
-5
0
5
10
Favours Non PR-AKI
Favours PR-AKI
