# Supplementary material for: Pregnancy outcomes in patients with acute kidney injury during pregnancy: a systematic review and meta-analysis
Source: BMC Pregnancy Childbirth. 2017 Jul 18;17:235. doi: 10.1186/s12884-017-1402-9 (PMC5516395; doi:10.1186/s12884-017-1402-9)
Supplement: Supplementary file 3 — Hazard ratios of cesarean delivery for pregnant women with versus without acute kidney injury. (PPTX 68 kb) [file 12884_2017_1402_MOESM3_ESM.pptx]

## Slide 1
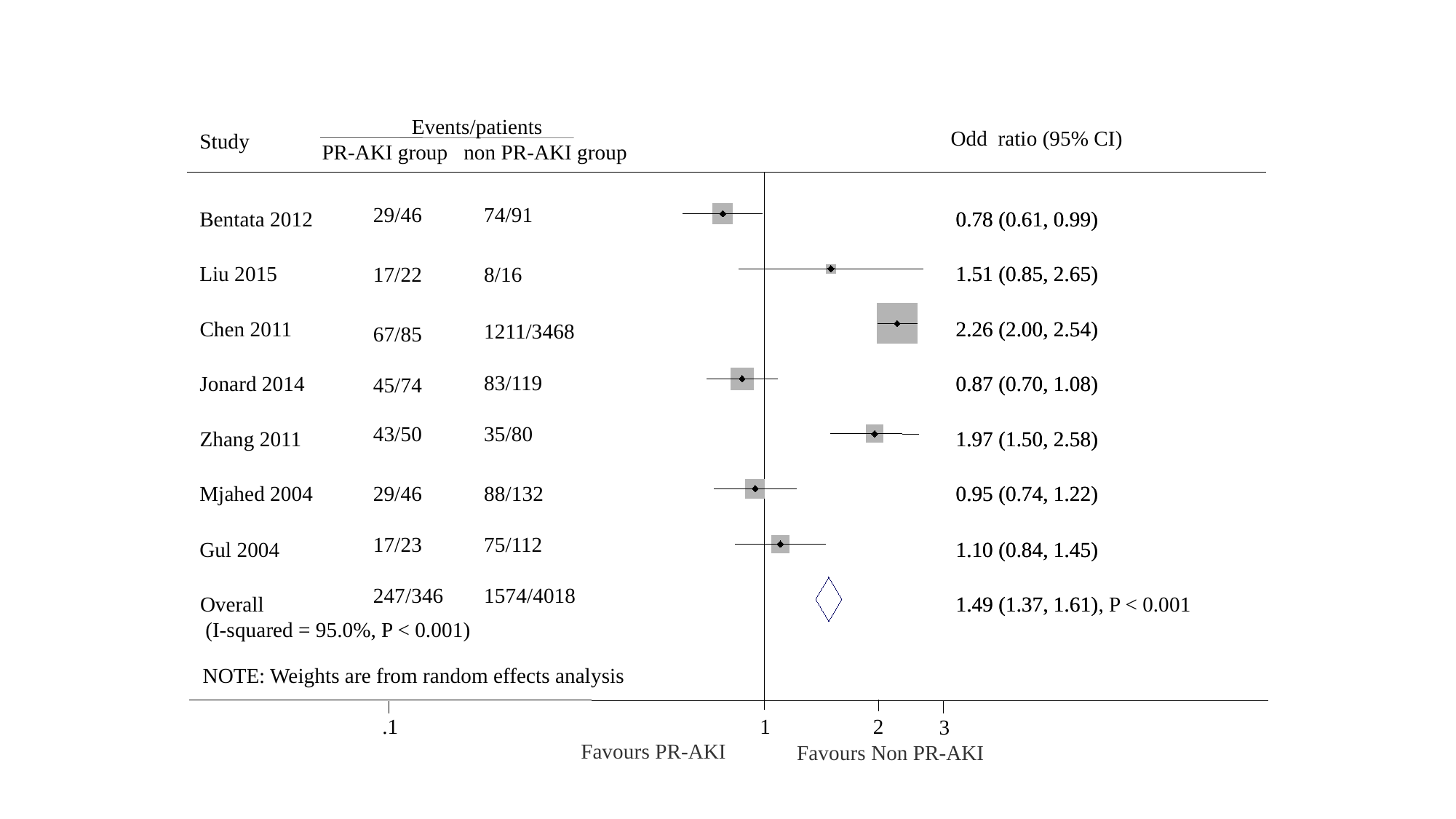

Events/patients
PR-AKI group non PR-AKI group
Odd ratio (95% CI)
Study
29/46
74/91
Bentata 2012
0.78 (0.61, 0.99)
0.78 (0.61, 0.99)
17/22
8/16
Liu 2015
1.51 (0.85, 2.65)
1.51 (0.85, 2.65)
1211/3468
Chen 2011
67/85
2.26 (2.00, 2.54)
2.26 (2.00, 2.54)
83/119
45/74
Jonard 2014
0.87 (0.70, 1.08)
0.87 (0.70, 1.08)
43/50
35/80
Zhang 2011
1.97 (1.50, 2.58)
1.97 (1.50, 2.58)
29/46
88/132
Mjahed 2004
0.95 (0.74, 1.22)
0.95 (0.74, 1.22)
17/23
75/112
Gul 2004
1.10 (0.84, 1.45)
1.10 (0.84, 1.45)
247/346
1574/4018
Overall
 (I-squared = 95.0%, P < 0.001)
1.49 (1.37, 1.61)
1.49 (1.37, 1.61), P < 0.001
NOTE: Weights are from random effects analysis
.1
1
2
3
Favours PR-AKI
Favours Non PR-AKI
