# Supplementary material for: Pregnancy outcomes in patients with acute kidney injury during pregnancy: a systematic review and meta-analysis
Source: BMC Pregnancy Childbirth. 2017 Jul 18;17:235. doi: 10.1186/s12884-017-1402-9 (PMC5516395; doi:10.1186/s12884-017-1402-9)
Supplement: Supplementary file 4 — Hazard ratios of cesarean delivery for pregnant women with versus without acute kidney injury by country. (PPTX 73 kb) [file 12884_2017_1402_MOESM4_ESM.pptx]

## Slide 1
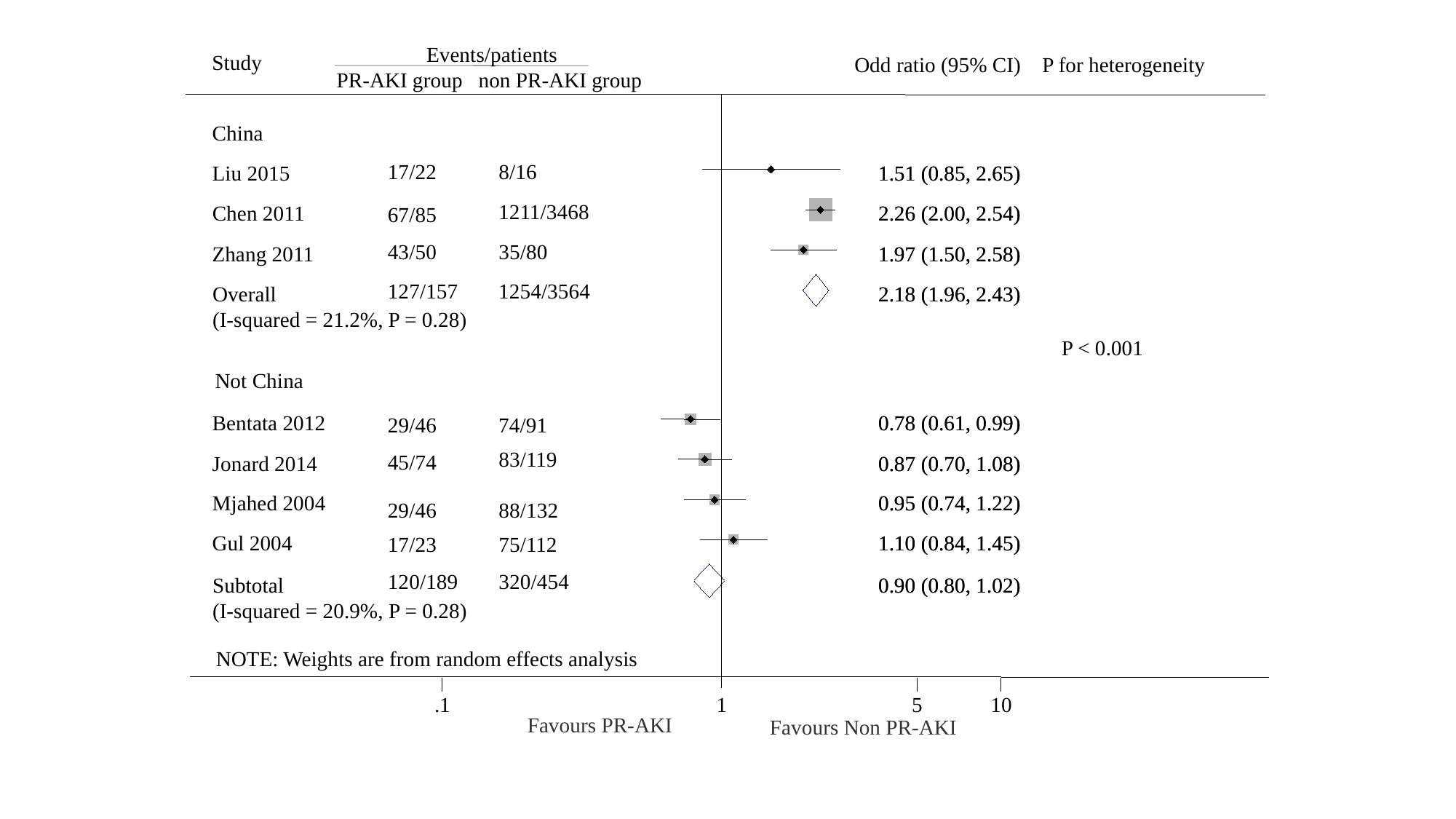

Events/patients
PR-AKI group non PR-AKI group
Study
Odd ratio (95% CI)
P for heterogeneity
China
17/22
8/16
Liu 2015
1.51 (0.85, 2.65)
1.51 (0.85, 2.65)
1211/3468
67/85
Chen 2011
2.26 (2.00, 2.54)
2.26 (2.00, 2.54)
43/50
35/80
Zhang 2011
1.97 (1.50, 2.58)
1.97 (1.50, 2.58)
127/157
1254/3564
Overall
(I-squared = 21.2%, P = 0.28)
2.18 (1.96, 2.43)
2.18 (1.96, 2.43)
P < 0.001
Not China
29/46
74/91
Bentata 2012
0.78 (0.61, 0.99)
0.78 (0.61, 0.99)
83/119
45/74
Jonard 2014
0.87 (0.70, 1.08)
0.87 (0.70, 1.08)
Mjahed 2004
0.95 (0.74, 1.22)
0.95 (0.74, 1.22)
29/46
88/132
17/23
75/112
Gul 2004
1.10 (0.84, 1.45)
1.10 (0.84, 1.45)
120/189
320/454
Subtotal
(I-squared = 20.9%, P = 0.28)
0.90 (0.80, 1.02)
0.90 (0.80, 1.02)
NOTE: Weights are from random effects analysis
.1
1
5
10
Favours PR-AKI
Favours Non PR-AKI
