# Supplementary material for: Pregnancy outcomes in patients with acute kidney injury during pregnancy: a systematic review and meta-analysis
Source: BMC Pregnancy Childbirth. 2017 Jul 18;17:235. doi: 10.1186/s12884-017-1402-9 (PMC5516395; doi:10.1186/s12884-017-1402-9)
Supplement: Supplementary file 5 — Hazard ratios of eclampsia, HELLP syndrome, placental abruption, and DIC for pregnant women with versus without acute kidney injury. (PPTX 92 kb) [file 12884_2017_1402_MOESM5_ESM.pptx]

## Slide 1
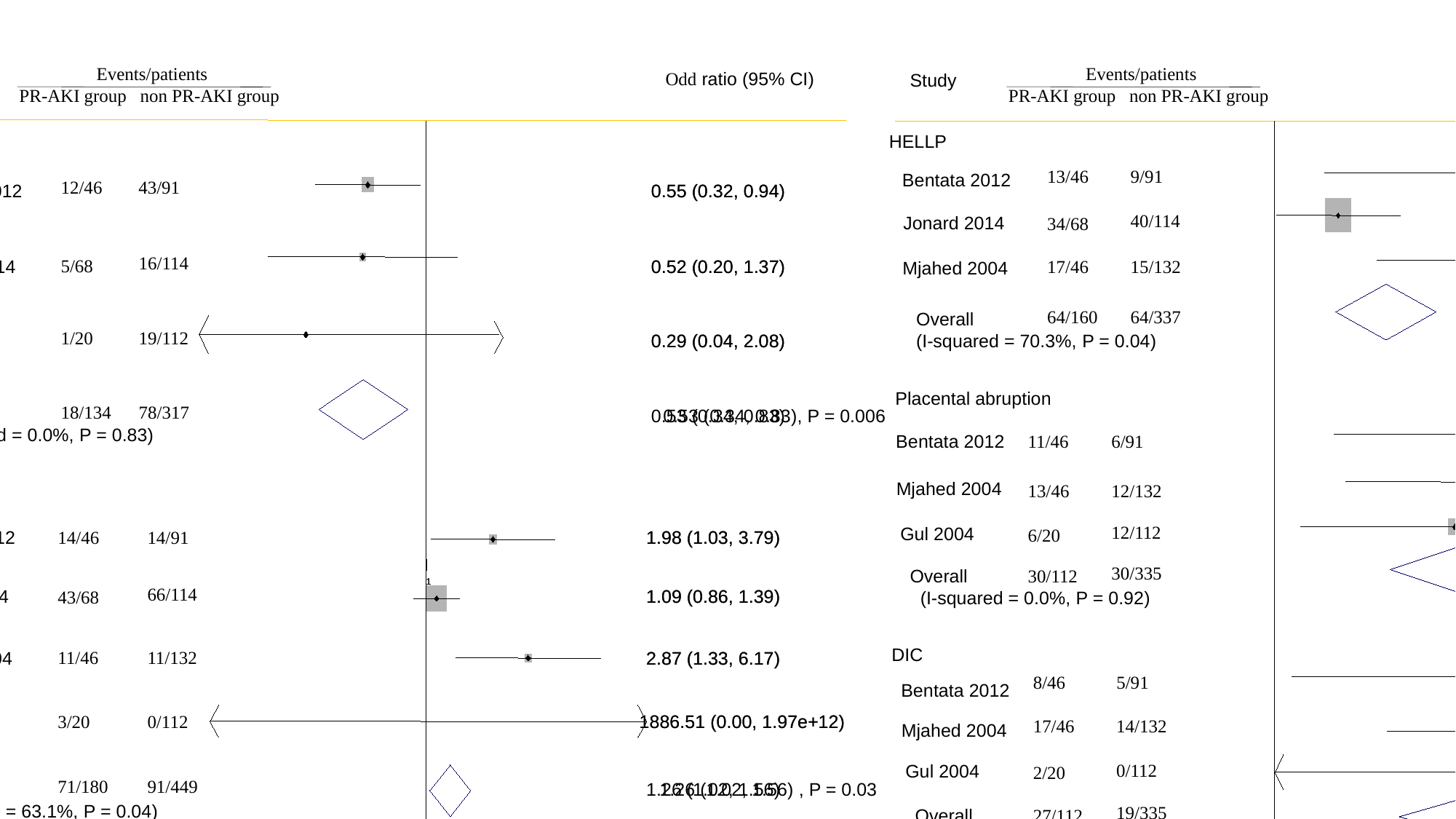

Events/patients
PR-AKI group non PR-AKI group
 Events/patients
PR-AKI group non PR-AKI group
Study
Odd ratio (95% CI)
Odd ratio (95% CI)
Study
HELLP
9/91
13/46
Bentata 2012
2.86 (1.32, 6.19)
2.86 (1.32, 6.19)
40/114
34/68
Jonard 2014
1.42 (1.01, 2.01)
1.42 (1.01, 2.01)
17/46
15/132
Mjahed 2004
3.25 (1.77, 5.97)
3.25 (1.77, 5.97)
64/160
64/337
Overall
(I-squared = 70.3%, P = 0.04)
1.86 (1.41, 2.46)
1.86 (1.41, 2.46) , P < 0.001
11/46
6/91
Bentata 2012
3.63 (1.43, 9.19)
3.63 (1.43, 9.19)
13/46
12/132
Mjahed 2004
3.11 (1.53, 6.32)
3.11 (1.53, 6.32)
12/112
6/20
Gul 2004
2.80 (1.19, 6.60)
2.80 (1.19, 6.60)
30/335
30/112
Overall
 (I-squared = 0.0%, P = 0.92)
3.13 (1.96, 5.02)
3.13 (1.96, 5.02) , P < 0.001
3.17 (1.10, 9.13)
3.17 (1.10, 9.13)
8/46
5/91
Bentata 2012
17/46
14/132
Mjahed 2004
3.48 (1.87, 6.49)
3.48 (1.87, 6.49)
0/112
2/20
1259.54 (0.00, 1.34e+12)
Gul 2004
19/335
27/112
Overall
(I-squared = 0.0%, P = 0.85)
3.41 (2.00, 5.84)
3.41 (2.00, 5.84), P < 0.001
NOTE: Weights are from random effects analysis
1
1
5
10
Favours Non PR-AKI
Favours PR-AKI
Eclampsia
12/46
43/91
Bentata 2012
0.55 (0.32, 0.94)
0.55 (0.32, 0.94)
16/114
5/68
Jonard 2014
0.52 (0.20, 1.37)
0.52 (0.20, 1.37)
1/20
19/112
Gul 2004
0.29 (0.04, 2.08)
0.29 (0.04, 2.08)
Placental abruption
18/134
78/317
Overall
(I-squared = 0.0%, P = 0.83)
0.53 (0.34, 0.83)
0.53 (0.34, 0.83), P = 0.006
Hemorrhage
14/46
14/91
Bentata 2012
1.98 (1.03, 3.79)
1.98 (1.03, 3.79)
1
1
66/114
43/68
Jonard 2014
1.09 (0.86, 1.39)
1.09 (0.86, 1.39)
11/46
11/132
DIC
Mjahed 2004
2.87 (1.33, 6.17)
2.87 (1.33, 6.17)
3/20
0/112
Gul 2004
1886.51 (0.00, 1.97e+12)
1886.51 (0.00, 1.97e+12)
71/180
91/449
Overall
(I-squared = 63.1%, P = 0.04)
1.26 (1.02, 1.56)
1.26 (1.02, 1.56) , P = 0.03
NOTE: Weights are from random effects analysis
.1
1
1
5
10
Favours PR-AKI
Favours Non PR-AKI
