# Supplementary material for: Pregnancy outcomes in patients with acute kidney injury during pregnancy: a systematic review and meta-analysis
Source: BMC Pregnancy Childbirth. 2017 Jul 18;17:235. doi: 10.1186/s12884-017-1402-9 (PMC5516395; doi:10.1186/s12884-017-1402-9)
Supplement: Supplementary file 6 — Gestational age at delivery in pregnant women with versus without acute kidney injury. (PPTX 84 kb) [file 12884_2017_1402_MOESM6_ESM.pptx]

## Slide 1
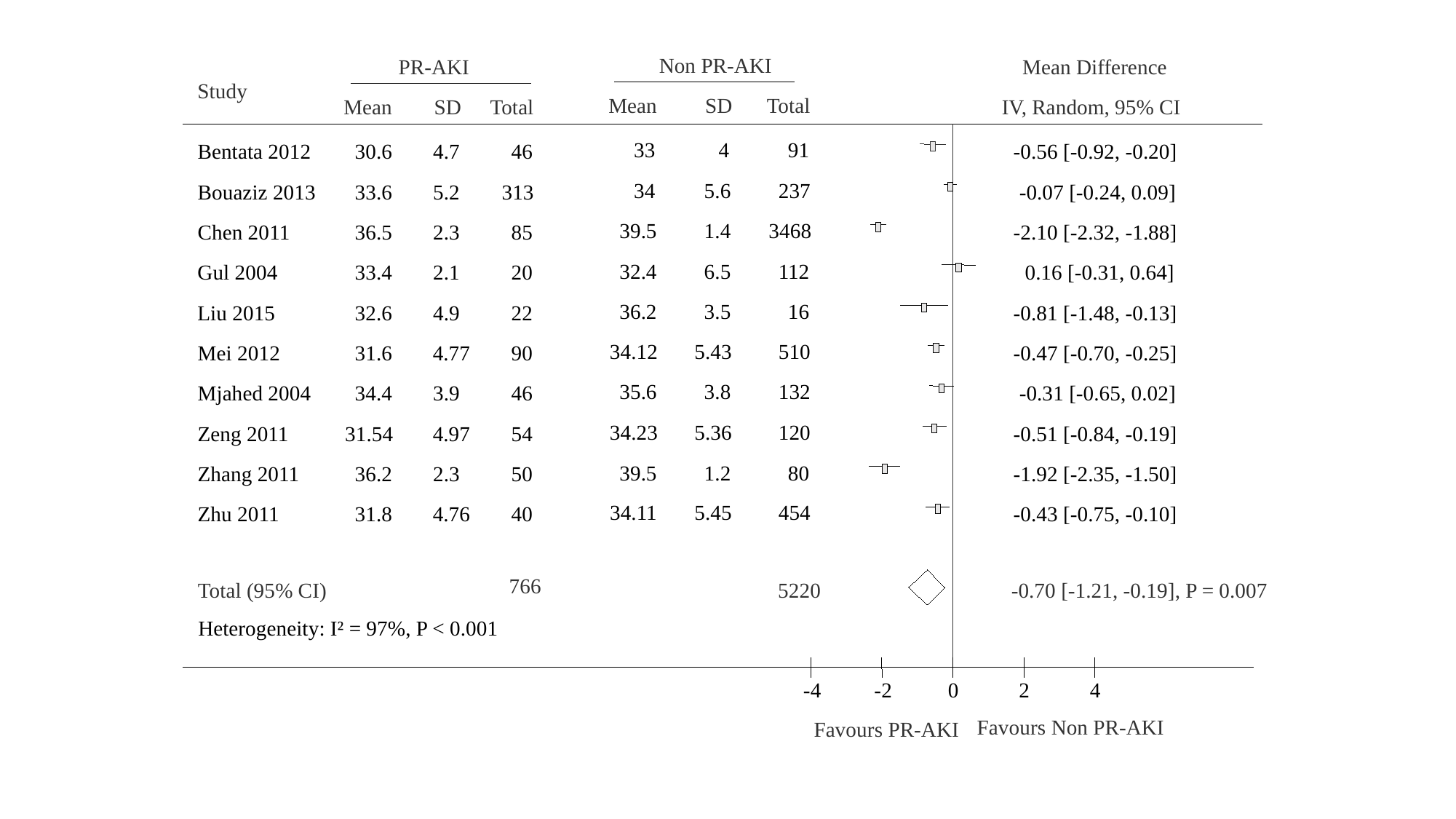

Non PR-AKI
PR-AKI
Mean Difference
Study
Mean
SD
Total
Mean
SD
Total
IV, Random, 95% CI
33
4
91
Bentata 2012
30.6
4.7
46
-0.56 [-0.92, -0.20]
34
5.6
237
Bouaziz 2013
33.6
5.2
313
-0.07 [-0.24, 0.09]
39.5
1.4
3468
Chen 2011
36.5
2.3
85
-2.10 [-2.32, -1.88]
32.4
6.5
112
Gul 2004
33.4
2.1
20
0.16 [-0.31, 0.64]
36.2
3.5
16
Liu 2015
32.6
4.9
22
-0.81 [-1.48, -0.13]
34.12
5.43
510
Mei 2012
31.6
4.77
90
-0.47 [-0.70, -0.25]
35.6
3.8
132
Mjahed 2004
34.4
3.9
46
-0.31 [-0.65, 0.02]
34.23
5.36
120
Zeng 2011
31.54
4.97
54
-0.51 [-0.84, -0.19]
39.5
1.2
80
Zhang 2011
36.2
2.3
50
-1.92 [-2.35, -1.50]
34.11
5.45
454
Zhu 2011
31.8
4.76
40
-0.43 [-0.75, -0.10]
766
Total (95% CI)
5220
-0.70 [-1.21, -0.19], P = 0.007
Heterogeneity: I² = 97%, P < 0.001
-4
-2
0
2
4
Favours Non PR-AKI
Favours PR-AKI
