# Supplementary material for: Pregnancy outcomes in patients with acute kidney injury during pregnancy: a systematic review and meta-analysis
Source: BMC Pregnancy Childbirth. 2017 Jul 18;17:235. doi: 10.1186/s12884-017-1402-9 (PMC5516395; doi:10.1186/s12884-017-1402-9)
Supplement: Supplementary file 7 — Birth weight in pregnant women with versus without acute kidney injury. (PPTX 64 kb) [file 12884_2017_1402_MOESM7_ESM.pptx]

## Slide 1
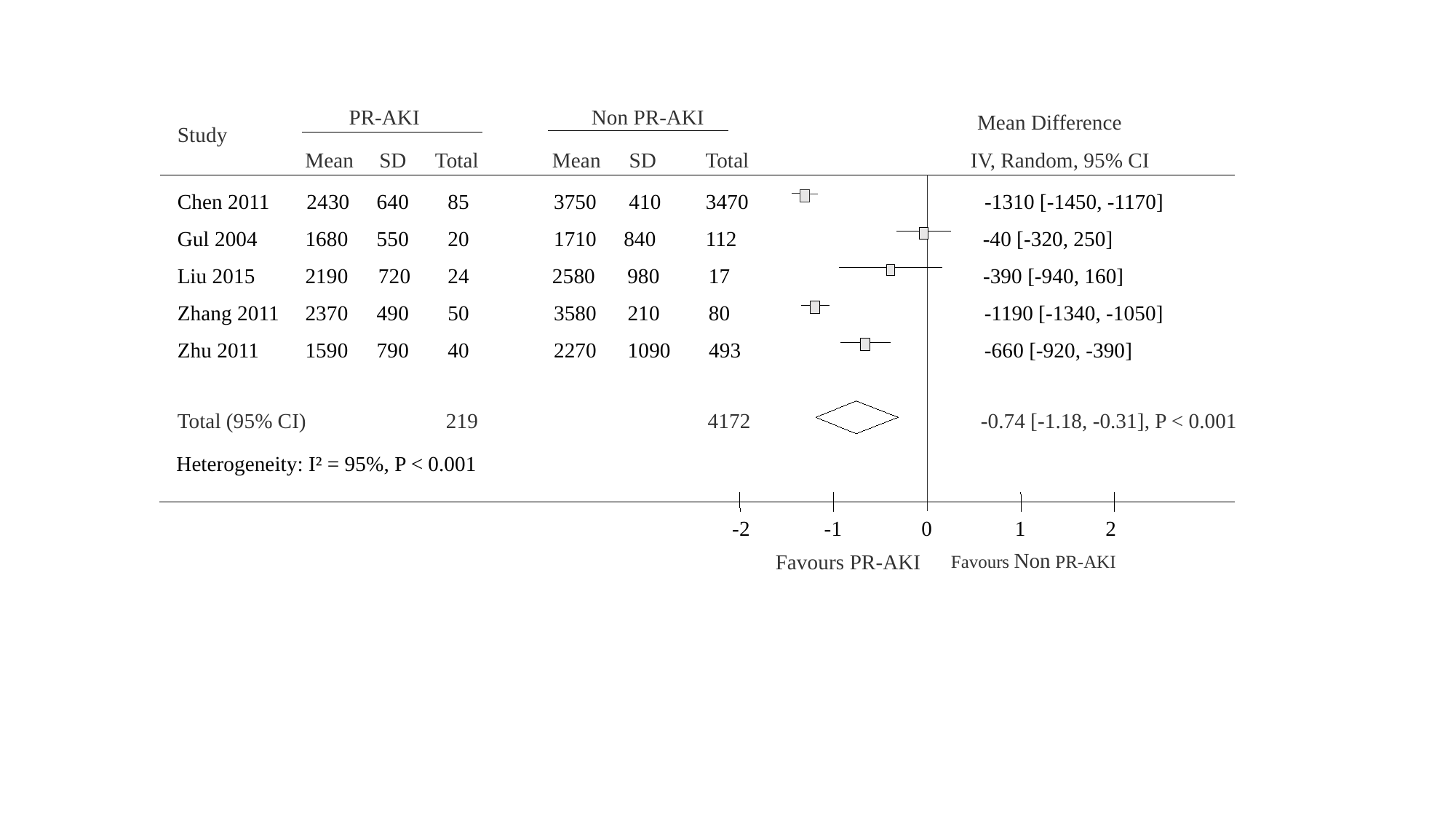

PR-AKI
Non PR-AKI
Mean Difference
Study
Mean
SD
Total
Mean
SD
Total
IV, Random, 95% CI
Chen 2011
2430
640
85
3750
410
3470
-1310 [-1450, -1170]
Gul 2004
1680
550
20
1710
840
112
-40 [-320, 250]
Liu 2015
2190
720
24
2580
980
17
-390 [-940, 160]
Zhang 2011
2370
490
50
3580
210
80
-1190 [-1340, -1050]
Zhu 2011
1590
790
40
2270
1090
493
-660 [-920, -390]
Total (95% CI)
219
4172
-0.74 [-1.18, -0.31], P < 0.001
Heterogeneity: I² = 95%, P < 0.001
-2
-1
0
1
2
Favours Non PR-AKI
Favours PR-AKI
