# Supplementary material for: Pregnancy outcomes in patients with acute kidney injury during pregnancy: a systematic review and meta-analysis
Source: BMC Pregnancy Childbirth. 2017 Jul 18;17:235. doi: 10.1186/s12884-017-1402-9 (PMC5516395; doi:10.1186/s12884-017-1402-9)
Supplement: Supplementary file 9 — Funnel plot with pseudo 95% confidence limits of maternal mortality among included studies. (PPTX 42 kb) [file 12884_2017_1402_MOESM9_ESM.pptx]

## Slide 1
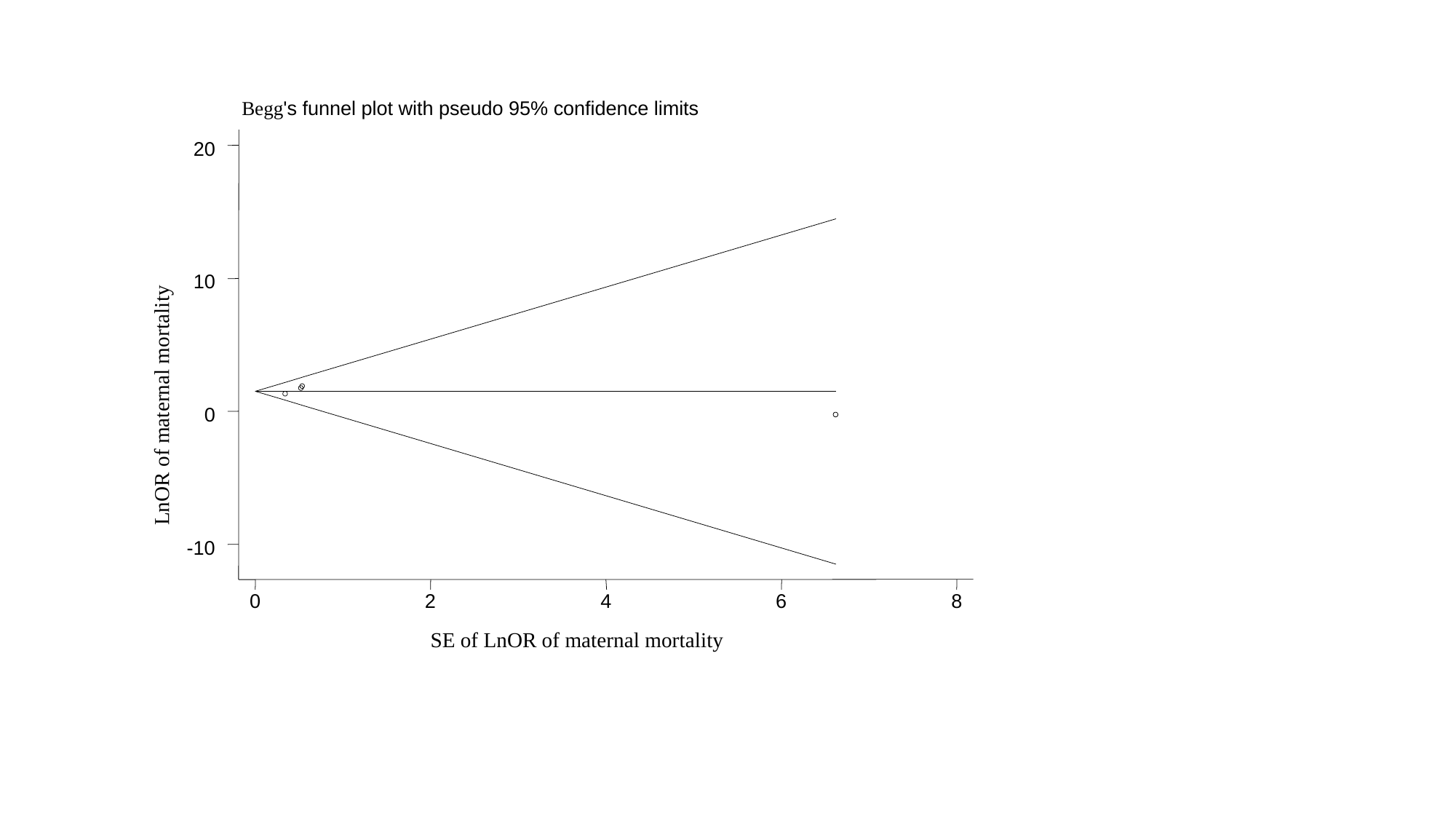

Begg's funnel plot with pseudo 95% confidence limits
20
10
LnOR of maternal mortality
0
-10
0
2
4
6
8
SE of LnOR of maternal mortality
