# Supplementary material for: Pregnancy outcomes in patients with acute kidney injury during pregnancy: a systematic review and meta-analysis
Source: BMC Pregnancy Childbirth. 2017 Jul 18;17:235. doi: 10.1186/s12884-017-1402-9 (PMC5516395; doi:10.1186/s12884-017-1402-9)
Supplement: Supplementary file 10 [file 12884_2017_1402_MOESM8_ESM.docx]

Table S1. Grade quality of evidence.

Design			Quality assessment					
Outcome	Experiment	Control	Publication bias	Inconsistency	Indirectness	Imprecision	No. of eligible studies	Quality
Cesarean delivery	PR-AKI group	non PR-AKI group	No	Yes	No	No	7	Very Low
Hemorrhage	PR-AKI group	non PR-AKI group	No	No	No	Yes	4	Very Low
HELLP syndrome	PR-AKI group	non PR-AKI group	No	No	No	Yes	3	Very Low
Placental abruption	PR-AKI group	non PR-AKI group	No	No	No	Yes	3	Very Low
Maternal death	PR-AKI group	non PR-AKI group	No	Yes	No	Yes	4	Very Low
DIC	PR-AKI group	non PR-AKI group	No	No	No	Yes	3	Very Low
Eclampsia	PR-AKI group	non PR-AKI group	No	No	No	Yes	3	Very Low
Fetal loss	PR-AKI group	non PR-AKI group	No	No	No	No	8	Low

Abbreviations: PR-AKI, Pregnancy related-Acute kidney injury; HELLP, hemolysis, elevated liver enzymes, and low platelets; DIC, disseminated intravascular coagulation.
